# The WOMAN trial: clinical and contextual factors surrounding the deaths of 483 women following post-partum haemorrhage in developing countries

**DOI:** 10.1186/s12884-020-03091-8

**Published:** 2020-07-16

**Authors:** Roberto Picetti, Lori Miller, Haleema Shakur-Still, Tracey Pepple, Danielle Beaumont, Eni Balogun, Etienne Asonganyi, Rizwana Chaudhri, Mohamed El-Sheikh, Bellington Vwalika, Sabaratnam Arulkumaran, Ian Roberts, on behalf of the WOMAN trial collaborators

**Affiliations:** 1 Clinical Trials Unit, London School of Hygiene & Tropical Medicine, Keppel Street, London, WC1E 7HT UK; 2 Maternity Unit, Kumba District Hospital, Kumba, Southwest Province Cameroon; 3 Holy Family Hospital, Gynaecology & Obstetrics Unit 1, F-762 Said Pur Road, Satellite Town, Rawalpindi, Pakistan; 4 Department of Obstetrics and Gynaecology, Faculty of Medicine, University of Khartoum, Khartoum, Sudan; 5 Department of Obstetrics and Gynaecology, School of Medicine, University of Zambia, Lusaka, Zambia; 6 St George’s University of London, Room 1.126, First Floor, Jenner Wing, Cranmer Terrace, London, SW17 0RE UK

## Abstract

**Background:**

Post-partum haemorrhage (PPH) is a leading cause of maternal death worldwide. The WOMAN trial assessed the effects of tranexamic acid (TXA) on death and surgical morbidity in women with PPH. The trial recorded 483 maternal deaths. We report the circumstances of the women who died.

**Methods:**

The WOMAN trial recruited 20,060 women with a clinical diagnosis of PPH after a vaginal birth or caesarean section. We randomly allocated women to receive TXA or placebo. When a woman died, we asked participating clinicians to report the cause of death and to provide a short narrative of the events surrounding the death. We collated and edited for clarity the narrative data.

**Results:**

Case fatality rates were 3.0% in Africa and 1.7% in Asia. Nearly three quarters of deaths were within 3 h of delivery and 91% of these deaths were from bleeding. Women who delivered outside a participating hospital (12%) were three times more likely to die (OR = 3.12, 95%CI 2.55–3.81) than those who delivered in hospital. Blood was often unavailable due to shortages or because relatives could not afford to buy it. Clinicians highlighted late presentation, maternal anaemia and poor infrastructure as key contributory factors.

**Conclusions:**

Although TXA use reduces bleeding deaths by almost one third, mortality rates similar to those in high income countries will not be achieved without tackling late presentation, maternal anaemia, availability of blood for transfusion and poor infrastructure.

## Background

Post-partum haemorrhage (PPH), the leading cause of maternal death worldwide, accounts for about 19.7% of maternal deaths [1, 2]. There are considerable variations across regions with for instance PPH accounting for about 8.0% of maternal deaths in developed countries compared to 19.7% in the developing countries [1]. Maternal outcomes for obstetric haemorrhages, e.g. PPH, are also more severe in developing countries [3]. This disparity between developed and developing areas may be explained by the limited resources in the latter. Some of the deficiencies in the health systems include insufficient availability of properly formed birth attendants both in hospitals and to assist home births, insufficient emergency transport to a facility with better expertise, and limited availability of blood products and blood banks [4].

The WOMAN Trial was a large, international, randomised trial to assess the effects of tranexamic acid (TXA) on death and surgical interventions in women with PPH [5]. In 2017, we reported that early administration of TXA reduces death due to bleeding after PPH by about one third with no evidence of adverse effects [5].

Because TXA is a haemostatic drug, we hoped that it would reduce death from bleeding, but we did not expect any reduction in deaths unrelated to bleeding (e.g. from infection or eclampsia) [6]. For this reason, we recorded information on the cause of death in the WOMAN trial. Although assigning cause requires judgement, in a blinded clinical trial any inaccuracy will be unrelated to treatment allocation and should not cause bias. Nevertheless, to verify the accuracy of the cause of death, we asked doctors to provide a short narrative account of the events surrounding the death. In the WOMAN trial, 483 of the enrolled women died. The narratives of their deaths, although sometimes just a few lines written by the doctor who witnessed it, were helpful to shed some light on the circumstances of the women who died, revealing details otherwise not captured bythe trial data.

In this paper, we draw on these narratives to illuminate maternal death following a diagnosis of PPH. We hope that this will facilitate a deeper understanding of the risk factors for PPH death as well as of the circumstances of the women who die and of the doctors who care for them.

## Methods

The WOMAN trial was a randomised, placebo-controlled trial of the effect of TXA on death and maternal morbidity in women with PPH [5]. It included 20,060 women aged 16 years and older with a clinical diagnosis of PPH recruited from 193 hospitals in 21 countries between 2010 and 2016. Participating hospitals were selected on their ability to provide Comprehensive Emergency Obstetric Care, i.e. hospital care provision had to include parenteral administration of antibiotics, uterotonic drugs and parenteral anticonvulsants, manual removal of placenta, removal of retained products, performance of assisted vaginal delivery and neonatal resuscitation, blood transfusion and caesarean section services [7]. The fundamental eligibility criteria for women recruited into the trial were a diagnosis of PPH after a vaginal birth or caesarean section and the clinician’s uncertainty about whether to use TXA in particular a woman with PPH [5, 8]. The clinical diagnosis of PPH could be based on the clinical estimation of blood loss. After confirming eligibility and completing the consent procedures, we collected baseline data on the trial entry form completed just before randomisation. We randomly allocated women to receive an intravenous injection of 1 g of TXA or matching placebo by selection of a blinded treatment pack. If bleeding continued after 30 min or restarted within 24 h of the first dose, we gave a second dose of 1 g of TXA or placebo. We have published a detailed account of the trial rationale, design, eligibility criteria, methods, results, and ethics committee approvals elsewhere [5, 8]. A written informed consent was obtained for each participant according to the procedures detailed in Shakur et al. [8]. The study was approved by the UK National Research Ethics Service (Berkshire Research Ethics Committee reference number 10/H0505/111) and by the London School of Hygiene and Tropical Medicine Ethics Committee (reference number 5536).

We recorded outcome data at death, discharge or 6 weeks (42 days) after randomisation (whichever occurred first). We obtained follow-up data for 99.8% of women. Doctors sent the data to the coordinating centre by direct entry into an electronic database, by fax or via encrypted data forms (sent by email, or uploaded to a secure server). We monitored adherence to the protocol and data quality by central monitoring, statistical data checking, and site visits, during which we conducted source data verification.

Whenever a woman died, we asked the obstetrician to record the immediate cause of the death (the final pathophysiological process leading to death). If there was more than one immediate cause, we asked for the most important. We also asked for a short narrative account of the events surrounding the death. The chief investigator and project director reviewed the narratives and if they appeared inconsistent with the given cause of death, we asked the obstetrician to review the cause of death (although in each case the judgement of the obstetrician prevailed). In most cases, a post-mortem examination was not conducted due to cultural or other local reasons. Narratives were reviewed to identify if contributing factors were mentioned in relation to the deaths. The qualitative narratives were managed in NVivo 11 (released 2015, QSR International) and cause of death and contributing factors were identified using a thematic analysis. We edited the narrative data to correct spelling and punctuation and we removed any identifying information related to the participant. Quotes are identified by numbers referring to their positions in the supplementary material (Table S1).

We conducted univariable analyses to describe risk factors for death and the characteristics of the study population that were collected from the trial entry forms. We conducted statistical analysis using Stata Statistical Software: Release 15 (College Station, TX: StataCorp LLC).

## Results

Most of the 20,060 trial participants were recruited in Africa (12,343) and Asia (6030). There were 483 maternal deaths with case fatality rates of 3.0% (375 women) and 1.7% (105 women) in Africa and Asia, respectively. In Europe, 1049 women were recruited and none died. We obtained narratives for 52% of the women who died.

We show the baseline characteristics of the 483 women who died in Table 1. The odds of death increased with increasing age. The odds of death for mothers with stillbirths was five times higher than for those with live infants. Over one third of the women who died (35.1%) gave birth to a stillborn baby, compared to less than one tenth (9.3%) of those who survived. High estimated blood loss (> 1000 mL), hypotension (systolic blood pressure < 90 mmHg) and haemodynamic instability (based on clinical signs such as low blood pressure, tachycardia, falling urine output that require interventions such as the administration of intravenous fluids) were strongly associated with the risk of death. Within 3 h from delivery, 13.3% of the 483 women died, and an additional 59.8% died between 3 and 24 h post-delivery. The maximum survival time was 28.7 days.

**Table 1:** Baseline characteristics and stillbirths of women in the WOMAN trial who survived, and of the women who died. The second and third columns report the number of women in each category and the corresponding percentage. The fourth and fifth columns report the odds ratios, and the *P* values calculated from the likelihood ratio test

	n (%) alive	n (%) dead	OR (95% CI)	*P*-value
**Age at randomisation (years)**				
≤ 25	6738 (34.49)	104 (21.53)	1	< 0.001
26–35	10,703 (54.78)	284 (58.80)	1.72 (1.37–2.16)	
≥ 36	2090 (10.70)	95 (19.67)	2.94 (2.22–3.91)	
Missing	7 (0.04)	0.0		
**Baby delivered in hospital**				
Yes	17,249 (88.28)	341 (70.60)	1	< 0.001
No	2287 (11.71)	141 (29.19)	3.12 (2.55–3.81)	
Missing	2 (0.01)	1 (0.21)		
**Type of delivery**				
Vaginal	13,871 (70.99)	320 (66.25)	1	0.03
Caesarean section	5663 (28.98)	162 (33.54)	1.24 (1.02–1.50)	
Missing	4 (0.02)	1 (0.21)		
**Systolic BP (mmHg)**				
< 90	3560 (18.22)	276 (57.14)	6.13 (5.05–7.45)	< 0.001
90–120	13,446 (68.82)	170 (35.20)	1	
121–140	1775 (9.08)	25 (5.18)	1.11 (0.73–1.70)	
> 140	752 (3.85)	12 (2.48)	1.26 (0.70–2.28)	
Missing	5 (0.03)	0.0		
**Estimated volume of blood lost (mL)**				
≤ 1000	10,327 (52.86)	77 (15.94)	1	< 0.001
1001–2000	8072 (41.31)	213 (44.10)	3.54 (2.72–4.60)	
> 2000	1137 (5.82)	193 (39.96)	22.77 (17.36–29.86)	
Missing	2 (0.01)	0.0		
**Haemodynamic instability**				
No	8168 (41.81)	26 (5.38)	1	< 0.001
Yes	11,369 (58.19)	457 (94.62)	12.63 (8.50–18.77)	
Missing	1 (0.01)	0.0		
**Primary cause of haemorrhage**				
Uterine atony	12,504 (64.00)	257 (53.21)	1	< 0.001
Placenta praevia/accreta	1835 (9.39)	40 (8.28)	1.06 (0.76–1.49)	
Surgical trauma/tears	3582 (18.33)	99 (20.50)	1.34 (1.06–1.70)	
Other/Unknown	1617 (8.28)	87 (18.01)	2.62 (2.04–3.36)	
**Stillbirths**				
No	17,716 (90.67)	313 (64.80)	1	< 0.001
Yes	1817 (9.30)	169 (34.99)	5.26 (4.34–6.39)	
Missing	5 (0.03)	1 (0.21)		

Although most of the trial participants (88.0%) gave birth in a participating hospital, 12.0% gave birth elsewhere and were brought to the hospital for emergency care. These women were over three times more likely to die (OR = 3.12, 95%CI 2.55–3.81). Of the women who gave birth elsewhere, 38.8% had a blood pressure below 90 mmHg, and 79.8% showed signs of haemodynamic instability, compared to 16.5 and 56.2% of those who delivered in hospital, respectively. Blood transfusions were given to 79.0% of women who did not deliver in a hospital compared to 51.4% of those who did. Of the women who delivered outside a hospital, 5.8% died compared with 1.9% of those who delivered in hospital.

*“The mother was referred from another health unit where she had delivered and bled profusely. She only stayed with us for about 40 minutes and died during resuscitation before she was transfused.” (quote 162,* Table S1*)*

*“The patient was an un-booked gravida 3 para 2, brought by a TBA [traditional birth attendant] from home with APH [ante-partum haemorrhage] and prolonged labour. Laparotomy revealed extensive uterine rupture and large amount of internal bleeding. She died on the theatre table while resuscitative efforts were still on-going.” (quote 156,* Table S1*)*

*“Following an unsupervised home delivery of a set of twins, the placentae of the babies were retained. All efforts to remove them at the facilities she got to were unsuccessful until she got to [named hospital], by which time we estimated she would have lost about 2000 mls of blood. Under halothane, another attempt at removal was made yielding only a ragged placenta and a possibly adherent second one. In view of the bleeding, the doctors proceeded to hysterectomy. However, she expired after 4 hours post operatively.” (quote 191,* Table S1*)*

Women sometimes arrived at hospital from other health facilities where they gave birth and were already in a critical condition upon arrival at the hospital.

*“Delivered vaginally in a private clinic to a stillbirth. … Referred to our unit with post-partum haemorrhage. Admitted in our unit at midnight in shock, no recordable blood pressure, cold extremities, pre renal failure, gasping breath sounds, atonic uterus. Evacuated more than one litre of blood clots from her uterus. The amount of blood loss before admission not indicated in the referral note. Started on IV fluids ... Started on blood transfusion, bleeding stopped one hour later at 2am, uterus well contracted. Blood pressure improved to 90/50. Received 2 units of whole blood and 2 units of fresh frozen plasma. At 5am developed profuse vaginal bleeding with atonic uterus again, prepared for theatre, no more blood available in our blood bank, intubated but arrested before further treatment.” (quote 160,* Table S1*)*

*“The patient had an emergency Caesarean section performed in another health unit and was referred to [named hospital] hospital due to continued per vaginal bleeding- PPH. On arrival, she was in critical condition, unconscious with cardiovascular collapse. Her clothing was all soiled with blood and she had active per vaginal bleeding. There were no obvious signs of free peritoneal fluid. Uterus was at about 22 weeks.” (quote 181,* Table S1*)*

*“Patient underwent c/section at peripheral hospital and was referred to our hospital in state of shock. After resuscitating the patient, a laparotomy was arranged because patient did not respond to non-surgical interventions and there was worsening hemoperitonium. Surgery revealed that there was uterine atony and massive hemoperitonium, several uterotonic drugs and tranexamic acid was given. In the meantime, patient became hemodynamically unstable when hysterectomy was performed.” (quote 232,* Table S1*)*

Several of the death narratives highlighted the contribution of severe anaemia which complicated emergencies where blood for transfusions was not readily available.

*“Had induction of labour on account of intrauterine foetal death but suffered from PPH with severe anaemia. … She could only get a pint of blood and efforts to get more blood for further transfusion was on-going when she died.” (quote 126,* Table S1*)*

*“The main cause of death was the anaemia which became complicated by heart failure from which she never recovered. If we had been able to rescue her from the failure, we would have had enough time to treat the pneumonia.” (quote 114,* Table S1*)*

*“PPH is an emergency and usually many things are done simultaneously, … she died because of severe blood loss/preexisting anaemia and lack of more blood for transfusion.” (quote 168,* Table S1*)*

*“Delivered in an outside facility, admitted to our site post-partum. ... Reported to have sickle cell anaemia, but this could not be confirmed before she died. Relatives were unable to pay or donate for further blood transfusion.” (quote 170,* Table S1*)*

**Fig. 1:**
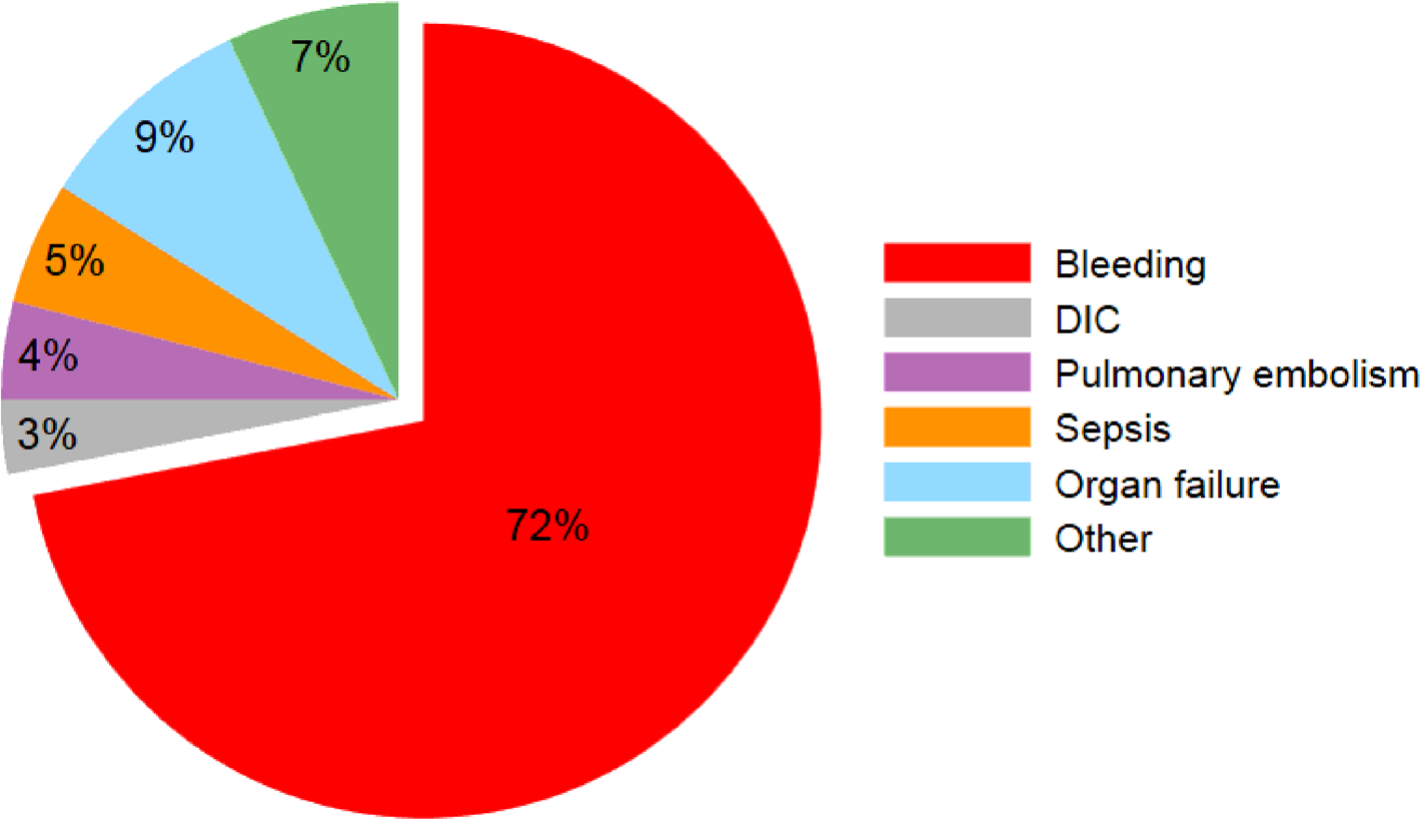
Causes of death for the 483 women who died in the WOMAN trail. The other causes included eclampsia, amniotic fluid embolism, anaesthetic complication, aspiration pneumonia, blood transfusion reaction, diabetic ketoacidosis, HELLP syndrome, uremic encephalopathy, and unknown causes

Among the women who died, 346 (72.0%) died from bleeding (Fig. 1). Of those who died within 3 h of delivery, 91.0% were due to bleeding. Of those who died within 24 h of delivery, 83.0% were due to bleeding. The primary cause of haemorrhage in most women was uterine atony, which cause the death of 53.2% of the women who died (Table 1). The primary cause of haemorrhage was recorded as “Other/Unknown” for 1704 women, 87 of whom died (OR = 2.62, 95%CI = 2.04–3.36).

The women who died lost on average 2.1 L (SD = 0.9 L, median = 2 L, IQR = 1.5–2.5 L), whereas those who lived lost on average 1.2 L of blood (SD = 0.6 L, median = 1 L, IQR = 0.8–1.5 L). Among the women who died 92.1% received a blood transfusion, whereas 53.8% of those who lived received one. Women who died received a mean of 4.4 units (SD = 3.4) of transfused blood. Blood was often unavailable, either because of blood shortages or because relatives could not afford to purchase it.

*“She delivered outside our hospital and was admitted in our facility haemorrhaging. She stopped bleeding after she was resuscitated but unfortunately, was not able to get blood transfused. Our hospital hardly gets voluntary blood donors. We get blood mainly from 'coerced' donors, i.e. relatives and caregivers of patients that require blood transfusion, surgery etc. Emergencies (like this patient) get blood if the blood bank is well stocked at the time or if they have funds (most of our patients do not, unfortunately) to source blood from private banks which we can transfuse. This woman's blood group was not available and she was unable to procure blood from elsewhere. She died because we could not replace all that she had lost (and I'm sure she had lost a great deal before she got to us).” (quote 130,* Table S1*)*

*“Admitted with cord prolapse. Had caesarean section to a fresh stillbirth. While in the recovery developed PPH which was controlled with oxytocin and misoprostol. Developed PPH again at 09:00hrs with uterine atony, same intervention initiated. Noted to be still bleeding by 10:15hrs, decision to take her back to theatre for hysterectomy made, transferred to theatre, actively bleeding, tried to get blood for blood transfusion but her blood type not available in our unit and regional blood transfusion centre. Mother died on the operating table at intubation before hysterectomy at 11:40hrs.” (quote 212,* Table S1*)*

*“Ruptured uterus was diagnosed during surgery; doctors proceeded to Caesarean hysterectomy. She suffered a first arrest during conversion from regional to general anaesthesia. Was resuscitated successfully. ... Haemostasis had been secured before 2nd cardiopulmonary arrest. CPR was performed till she was certified dead at 10:57. Patient had since stopped bleeding, but there was delayed replacement of lost blood (she had no money, and it took a while to get a credit deferment form). We think cause of death was a combination of allergic reaction to anaesthetic agents and blood loss.” (quote 172,* Table S1*)*

Although most deaths were from bleeding, 28.4% (137 women) were from non-bleeding causes of which organ failure and sepsis were the most common (Fig. 1). The “other” category includes deaths from eclampsia, amniotic fluid embolism, anaesthetic complication, aspiration pneumonia, blood transfusion reactions, diabetic ketoacidosis, HELLP syndrome (haemolysis, elevated liver enzyme levels, and low platelet levels) and uremic encephalopathy.

*“She had severe hypertension superimposed with pre-eclampsia, complicated by renal failure and cortical blindness. When she became poorly, the last assessment that was made before she died was of a blood transfusion reaction (from a transfusion which was discontinued, then found to be type-incompatible). That is the more probable cause of death.” (quote 111,* Table S1*)*

*“The patient was referred with PPH. Severe preeclampsia diagnosed in our hospital. Liver and kidney functions deranged severely. HELLP syndrome diagnosed. There was sign of stroke. Laparotomy done due to an impression of hematoma collection in the peritoneal space.” (quote 120,* Table S1*)*

*“She had Caesarean section on account of severe preeclampsia and developed postpartum haemorrhage from uterine atony afterwards. She … had uterotonics administered and transfused with 2 units of whole blood and active bleeding subsided … . However, she developed pulmonary oedema and subsequently died of respiratory failure. This was mainly a complication of the severe preeclampsia.” (quote 133,* Table S1*)*

*“Reading through the evidence, I would say the cause of death is overwhelming sepsis [ … ] Yes, sepsis definitely occurred. She had high-grade fever, tenderness over the uterus and antibiotics were procured irregularly (remember, it is pay-as-you-go around here).” (quote 116,* Table S1*)*

*“The patient died at 19:30hrs … , the bleeding stopped after administration of … but went into cardiopulmonary arrest while awaiting blood transfusion and died despite efforts to resuscitate her. The baby was severely asphyxiated and died shortly after birth.” (quote 164,* Table S1*)*

## Discussion

By preventing and treating PPH, we hope to avoid maternal deaths from PPH. To achieve this, the WHO has developed a set of recommendations for the prevention and treatment of PPH, focusing on medical and surgical interventions, and their implementation. Based on the WOMAN trial results [5], TXA was added to these recommendations [9].

With 20,060 patients and 483 maternal deaths, ours is one of the largest studies of women with PPH ever conducted. Because we conducted the WOMAN trial in countries where the burden of maternal death from PPH is highest, our findings are directly relevant to these countries. A potential limitation to our study is that we obtained narratives on 52% of the maternal deaths in the WOMAN trial. We selected the narratives shown because they provided context for the quantitative data about the women who died. The full set of narratives is available for review in the supplement (Table S1). Another limitation is that nearly half of the narratives (49%) came from one African country, which may be due to the high number of women recruited to the trial in that country. As a result, we cannot make clear inferences on the state of the health care in that country compared to others because the number of women recruited to the trial varied greatly among countries. Similarly, because only one high income country participated to the trial and the trial was not meant to compare health facilities in high income countries vs. low- and middle-income ones, we cannot draw conclusions on the effect of quality of care on mortality.

Although the risk of death was higher for women who delivered outside of hospital, we cannot draw inferences about the relative safety of home versus hospital delivery on the basis of this study. Another problem is the lack of records on the amount of blood that patients lost before the hospital admission, and that may have caused life-threatening consequences. The women who were transferred to hospital for urgent management would be the most severely bleeding patients and would not be a representative sample of all women who develop PPH. As a result of this selection bias we should not make any causal inference. In some low and middle-income countries, about 40% of women deliver at home [10] with only rudimentary transport in the event of an obstetric emergency. Although health workers attend most births, most cannot give intravenous drugs. Transport to hospital can take hours, and many women exsanguinate before arrival. Our data show that many women arrived in a critical condition and died soon after arrival. Early intravenous TXA reduces death from bleeding, but this is not an option for many thousands of women who deliver outside healthcare facilities. Intramuscular TXA has the potential to increase timely access to this life saving drug. Most health workers can give IM oxytocin and could give IM TXA if shown to be effective. The World Health Organization (WHO) has recommended that research into other routes of administration of TXA is a priority [9], and this research is in progress.

Many women do not deliver in hospitals because of costs, education or the perception or accessibility of health services [4, 11–16]. Studies on the factors that affect maternal mortality extensively used the three delays model examining barriers on the demand side (phases 1 and 2 delay) and on the supply side (phase 3 delay) [17] to understand and intervene on the barriers to prevent maternal death [4, 11–16].

According to the records, bleeding was the leading cause of death. Most women in our study received treatments aimed to prevent and stop blood loss, and, importantly, they also received blood transfusions (92.1%), which should help preventing death in case of major PPH [18]. However, some of the narratives revealed that sometimes the amount of blood transfused might not have been sufficient, or the required blood type was not available, or that the patient or her family could not afford to buy more blood units. Hence, in developing countries availability of blood for transfusion may be a factor that leads to maternal deaths. This agrees with other studies showing that poverty, lack of donors and logistic problems contribute to scarcity of safe blood in low- and middle-income countries [19–25]. However, our study cannot confirm that deficit of blood transfusion was a cause of death, nor that the transfusion was administered too late. The actual impact of safe blood availability on patients’ health, and how to address blood shortage should be the subject of further research in developing countries [24]. Research and interventions should target diverse areas such as increasing information to donors on safety of donations, the possibility of blood donations before delivery rather than after, financing steady blood availability within countries.

A little more than a quarter (28.4%) of the 483 women died of causes other than bleeding. Most of the women who died more than 24 h after giving birth (60.2%) died of causes other than bleeding, whereas among the women who died within 24 h from giving birth 85% of them died because of bleeding. This suggests a temporal shift in the causes of death, with bleeding being more important early on. The narratives show that these women had concurrent health complications that often lead to organ or respiratory failures, or death due to sepsis. In one narrative, sepsis, which caused the death of 5% of the 483 women, lead to the death of the patient because she could not buy the necessary antibiotics. In another case, the patient died because of she received a transfusion with the wrong blood type. To prevent transfusion errors, which result in harmful consequences to patients, waste of blood and money, new low-cost methods should be researched and developed to improve areas like patient identification, sample labelling or obtaining the correct blood type [24, 26–28].

The number of stillbirths was higher for the women who died than the women who lived. Based on our data, it is not possible to conclude the causal direction between stillbirths and the mothers’ complications and deaths. The issue around the causes of stillbirths is a problem at a worldwide level because of the poor quality of information, and the classification of causes is inconsistent between low-, middle-, and high-income countries [29, 30].

Several narratives highlighted the contribution of anaemia to maternal death. Although we did not measure haemoglobin on all women, a sub-study conducted at University College Hospital, Ibadan (Nigeria) [31–33], showed that most (88%) women were anaemic (haemoglobin < 110 g/L) and 40% were severely anaemic (haemoglobin < 70 g/L) at the time of PPH onset [31]. Women with such low haemoglobin levels have little physiological reserve and even mild to moderate bleeding can have serious consequences. Anaemia is caused by iron deficiency, micronutrients deficiency, infections, and disorders of haemoglobin synthesis, and is highly prevalent in Africa and South-East Asia [34]. Maternal anaemia increases the risk of PPH and the likelihood of severe morbidity or death should PPH occur [35, 36]. Blood shortages would exacerbate the risk from maternal anaemia. Several of the narratives highlighted the absence of blood for transfusion due to availability or cost. Poverty, lack of donors and logistic problems contribute importantly to blood shortages [19–25]. For women with severe anaemia, treatment of an established PPH is often too late to prevent death and effective prevention is needed. The WOMAN-2 trial will evaluate the effect of TXA for the prevention of PPH in high-risk anaemic women [37].

Although women who delivered outside of the hospital had a substantially higher case fatality, the case fatality of women who delivered in hospital was 1.9%. The data presented here highlight some of the factors that may account for this high hospital case fatality rate including the lack of timely intervention, inadequate infrastructure, maternal anaemia with limited access to safe blood transfusion and other important maternal co-morbidities that increase the risk of death. Whilst it seems unlikely that we will achieve maternal death rates comparable to those in high-income settings without addressing these structural issues, it is important to note that early administration of TXA reduced the risk of death due to bleeding by almost one third despite these constraints. Indeed, further analyses of the WOMAN trial data suggest that many women were so critically ill at the time of randomisation that their death was imminent and inevitable regardless of treatment [38]. This raises the possibility that if women are treated very soon after bleeding onset, as recommended by the WHO, the effect of TXA on reducing the risk of death due to bleeding may exceed that observed in the WOMAN trial.

## Conclusions

This study highlights factors contributing to death after PPH. Blood loss was the leading cause of death and the narratives emphasise the lack of blood for transfusion either from blood shortages or the inability to pay for it when available. This is particularly important for women with anaemia. Many women gave birth outside hospital and were transferred for treatment in a critical condition. These narratives, collected in the context of a randomised trial of tranexamic acid for women with PPH, underscore the contribution of the healthcare infrastructures to the unacceptably high maternal mortality from post-partum haemorrhage in many low and middle income settings.

## Supplementary information

**Additional file 1: Table S1.** Narratives dataset. Full set of narratives analysed in this study.

## Data Availability

The dataset containing the full list of narratives analysed in the current study are available as supplementary online material (Table S1). The same dataset is available on the Free Bank of Injury and Emergency Research Data (freeBIRD), administered by the LSHTM Clinical Trials Unit [https://freebird.lshtm.ac.uk/index.php?cID=158, free registration required].

## Abbreviations

HELLP: haemolysis, elevated liver enzyme levels, and low platelet levels
IM: intramuscular
IQR: Interquartile range
SD: Standard deviation
OR: Odds ratio
PPH: Post-partum haemorrhage
TXA: Tranexamic acid
